# High rate of pneumococcal bacteremia in a prospective cohort of older children and adults in an area of high HIV prevalence in rural western Kenya

**DOI:** 10.1186/1471-2334-10-186

**Published:** 2010-06-23

**Authors:** Daniel R Feikin, Geoffrey Jagero, Barrack Aura, Godfrey M Bigogo, Joseph Oundo, Bernard W Beall, Angela Karani, Susan Morpeth, M Kariuki Njenga, Robert F Breiman

**Affiliations:** 1 International Emerging Infections Program, Centers for Disease Control and Prevention, Mbagathi Road, off Mbagathi Way, Nairobi, Kenya; 2 Kenya Medical Research Institute/Centers for Disease Control, Research and Public Health Collaboration, P.O. Box 1578, Kisumu, Kenya; 3 Respiratory Diseases Branch, Division of Bacterial Diseases, Centers for Disease Control and Prevention, 1600 Clifton Rd. Atlanta, Georgia 30333; 4 KEMRI/Wellcome Trust Research Programme, Centre for Geographic Medicine Research - Coast, Kilifi, Kenya

## Abstract

**Background:**

Although causing substantial morbidity, the burden of pneumococcal disease among older children and adults in Africa, particularly in rural settings, is not well-characterized. We evaluated pneumococcal bacteremia among 21,000 persons ≥5 years old in a prospective cohort as part of population-based infectious disease surveillance in rural western Kenya from October 2006-September 2008.

**Methods:**

Blood cultures were done on patients meeting pre-defined criteria - severe acute respiratory illness (SARI), fever, and admission for any reason at a referral health facility within 5 kilometers of all 33 villages where surveillance took place. Serotyping of *Streptococcus pneumoniae *was done by latex agglutination and quellung reaction and antibiotic susceptibility testing was done using broth microdilution. We extrapolated incidence rates based on persons with compatible illnesses in the surveillance population who were not cultured. We estimated rates among HIV-infected persons based on community HIV prevalence. We projected the national burden of pneumococcal bacteremia cases based on these rates.

**Results:**

Among 1,301 blood cultures among persons ≥5 years, 52 (4%) yielded pneumococcus, which was the most common bacteria isolated. The yield was higher among those ≥18 years than 5-17 years (6.9% versus 1.6%, p < 0.001). The highest yield was for inpatients with SARI (10%), compared with SARI outpatients (3%) and acute febrile outpatients (1%). Serotype 1 pneumococcus was most common (42% isolates) and 71% were serotypes included in the 10-valent pneumococcal conjugate vaccine (PCV10). Non-susceptibility to beta-lactam antibiotics was low (<5%), but to trimethoprim-sulfamethoxazole was high (>95%). The crude rate of pneumococcal bacteremia was 129/100,000 person-years, and the adjusted rate was 419/100,000 person-years. Nineteen (61%) of 31 patients with HIV results were HIV-positive. The adjusted rate among HIV-infected persons was 2,399/100,000 person-years (Rate ratio versus HIV-negative adults, 19.7, 95% CI 12.4-31.1). We project 58,483 cases of pneumococcal bacteremia will occur in Kenyan adults in 2010.

**Conclusions:**

Pneumococcal bacteremia rates were high among persons ≥5 years old, particularly among HIV-infected persons. Ongoing surveillance will document if expanded use of highly-active antiretroviral treatment for HIV and introduction of PCV10 for Kenyan children (anticipated in late 2010) result in substantial secondary benefits by reducing pneumococcal disease in adults.

## Background

The burden of disease caused by *Streptococcus pneumoniae *(pneumococcus) in young African children is well-established [1-3]. A recent analysis estimated over 4 million cases and almost a half-million deaths from pneumococcal disease occur among African children < 5 years old annually [3]. The burden of pneumococcal disease in older children and adults in Africa, particularly in rural settings, is less well-characterized, although is likely substantial. Several studies in Africa have shown pneumococcus to be one of the principal causes of severe bacterial bloodstream infections in hospitalized adults, along with *Mycobacterium tuberculosis *and non-*typhi salmonellae *[4-11].

Few studies have assessed rates of pneumococcal disease because of lack of well-defined denominators in most African settings [6,7,12-15]. Moreover, no studies have adjusted rates based on health-seeking patterns among adults in Africa, where many sick adults do not attend health facilities. Better understanding of adult pneumococcal burden and epidemiology in African adults, including the serotype distribution and contributing role of HIV disease, will help predict, target and evaluate preventive strategies. We evaluated two years of population-based surveillance from rural western Kenya to better define the epidemiology and burden of pneumococcal bacteremia among older children and adults, and identify epidemiological factors that could potentially decrease this burden.

## Methods

### Site

CDC's International Emerging Infections Program and the Kenya Medical Research Institute (KEMRI) have conducted population-based, morbidity surveillance since late 2005 in Asembo in rural western Kenya. The surveillance population on July 1, 2007 included 21,420 persons ≥5 years old living in approximately 6,000 households. All participants who resided permanently in the area for 4 calendar months and had been registered in the KEMRI/CDC Demographic Surveillance System were eligible for participation in surveillance [16,17]. Malaria transmission is endemic, occurring year-round, with approximately 50% of sick children and 15% of sick adults having malaria positive blood smears in 2006-2008 [16]. The inhabitants are poor with over 66% of individuals living below the poverty line [18].

### Surveillance methods

All 33 enrolled villages are within 5 kilometers of the referral facility, Lwak Mission Hospital, which has both outpatient and inpatient capacity. Study participants receive free medical care at Lwak for most acute conditions, including all possible infectious diseases. Patients are examined and diagnosed by study-trained clinical officers (similar to physician's assistants). As part of surveillance, blood cultures are done on enrolled persons ≥5 years meeting one of three case definitions.

1. Severe acute respiratory illness (SARI), defined as cough or difficulty breathing and either temperature ≥38.0°C or oxygen saturation < 90% (all patients).

2. Acute febrile illness, defined as a temperature ≥38.0°C, without SARI or other obvious source (e.g. bloody diarrhea), irrespective of malaria blood smear result (first two patients per day regardless of severity or diagnosis).

3. All patients admitted for conditions unrelated to injury or obstetrics.

Scannable paper questionnaires are completed on all sick visits at Lwak, documenting symptoms, health-seeking, physical exam, diagnosis, treatment and outcome (TeleForm^®^, Cardiff™, California).

Three other Ministry of Health outpatient health facilities are in or near the surveillance area. Blood cultures are not done at these facilities and no data is collected on visits to these facilities on participants of the surveillance population. No private clinics are in the area. HIV care was available in Lwak and one other area clinic during this time (one day per week); however, in 2008 only approximately 26% and 17% of HIV-infected persons were taking cotrimoxazole prophylaxis and highly active antiretroviral therapy (KEMRI/CDC data).

Community interviewers visit enrolled households every two weeks to inquire about illnesses [17]. Symptoms are recorded and an abbreviated physical exam is done by trained field workers. Participants are asked if and where they sought health care. Using household visit data, we defined SARI as an episode with cough or difficulty breathing and reported fever and defined acute febrile illness as someone with reported fever without cough, difficulty breathing, or bloody diarrhea.

### Laboratory

Blood collected for culture was inoculated into commercially-produced blood culture bottles (BACTEC™ Aerobic PLUS™, Becton Dickinson, Belgium). Seven-to-ten ml of blood was inoculated in bottles, which remained at room temperature (approximately 30°C) for 2-6 hours before being incubated in an automated BACTEC 9050 for 4 days. Blood from bottles signaling a positive culture was gram-stained and sub-plated onto standard media for bacteria identification using routine microbiologic techniques [19,20]. *Streptococcus pneumoniae *was identified by colony morphology, optochin susceptibility and bile solubility. Isolates were stored at -70°C in 20% glycerol broth. Antimicrobial susceptibility patterns were determined using broth microdilution at the CDC Streptococcus laboratory (Atlanta, US), and minimum inhibitory concentrations for nonmeningitis breakpoints were used according to standard interpretative criteria [19]. Serotyping was performed using latex agglutination and quellung reaction by KEMRI/Wellcome Trust in Kilifi, Kenya (typing sera and reagents purchased from Statens Serum Institut, Copenhagen, Denmark) or the CDC Streptococcus laboratory (CDC typing sera and reagents).

HIV testing was performed as part of a home-based testing initiative in 2008 in which all persons ≥13 years in the surveillance area were offered HIV testing. Approximately 79% of eligible persons agreed to have an HIV test or reveal previously positive results (10,197 of 12,906 eligible, KEMRI/CDC data). Eligible persons were administered two parallel HIV rapid tests (Determine™, Abbott Laboratories, USA) and Bioline™ (Standard Diagnostics, Kyonggi-do, Korea) by trained counselors, with a tie-breaker test for discordant results (Uni-gold™, Trinity Biotech, Ireland). We assumed that a person's HIV status during home-based testing was the same throughout the study period. Participants without available HIV results had refused, had out-migrated, or had died before the home-based testing initiative. We did not do HIV-testing in the clinic on most patients during this period.

### Data analysis

Clinic visits, household visits, and laboratory test results, including HIV, were linked by common ID numbers. Data analyses were performed using SAS (version 9.1). Differences in proportions were assessed by chi-square tests and means by t-tests. Crude rates were calculated as the number of cases of pneumococcal bacteremia per 100,000 person-years. Each participating individual contributed person-time according to their dates of residence within the Demographic Surveillance System from October 1, 2006 - September 30, 2008 [16]. 95% confidence intervals were calculated for crude rates using Fisher's method (Computer Programs for Epidemiologists, PEPI, version 4.0x) and for extrapolated rates using the delta method [21].

Two adjustments were applied on crude rates. First, a multiplier was included for the percentage of persons visiting Lwak Hospital who met a criterion for blood culture and did not have a culture done. The reasons for not getting cultured included the clinician not recognizing that the patient met the case definition, the patient refusing the blood draw, febrile patients presenting after two febrile persons had already been sampled that day, or an inpatient having already received intravenous antibiotics before blood for culture could be taken. The percentage of uncultured patients was obtained separately for 4 age categories and for each indication for blood culturing. A second adjustment was made based on the percentage of persons identified with each of the indications for blood culture at the household visit who visited a clinic other than Lwak for that illness. We assumed that those who visited another clinic had a comparable severity and spectrum of etiologies to those that visited Lwak for the same syndrome.

To estimate the pneumococcal bacteremia rate in HIV-infected persons, we calculated the numerator by applying the same proportion of HIV-positivity among patients with pneumococcal bacteremia in whom HIV status was known to those whom the HIV status was unknown. Population prevalence of HIV by age group was obtained from the home-based HIV testing initiative in the surveillance population The HIV prevalence among 18-34 year olds was 19%, among 35-49 year olds was 28%, and among those ≥50 years was 6%. HIV prevalence rates were applied to the person-time calculated for each age category to obtain the person-years denominator for adults with and without HIV. Children and adolescents aged 5-17 years were excluded from the HIV-specific rate calculations as they were not consistently included in home-based HIV testing so denominator populations were not calculable. Rate ratios between HIV-positive and -negative individuals were calculated using Breslow's test for 95% confidence intervals.

We projected the number of pneumococcal bacteremias among persons ≥18 years old in Kenya in 2010. We used the extrapolated rates of pneumococcal bacteremia among HIV-positive and HIV-negative persons, the 2010 projected age- and sex-specific population of Kenya, and the age- and sex-specific HIV prevalence among all Kenyans from the 2007 Kenya AIDS Indicator Survey (KAIS) [22,23]. Because KAIS did not do HIV-testing on persons ≥65 years old, we assumed that all persons ≥65 years were HIV-negative for purposes of the projection.

### Ethical review

The protocol and written informed consent forms for both surveillance and home-based HIV testing were reviewed and approved by the Ethical Review Board of KEMRI and the Institutional Review Board of CDC.

## Results

During the study period, 16,322 patients ≥5 years visited Lwak Hospital -- 15,078 (92%) outpatients and 1,244 (8%) inpatients. Of these, 1,301 (8%) had blood cultures done, which was 47% of those meeting the indications for blood culture. Among the 1,301 cultures, 89 (6.8%) yielded true pathogens (excluding 33 contaminants, 2.5% of total cultures). Eight (0.6%) bottles were BACTEC alarm-positive, but culture-negative. A reported history of prior antibiotic use for the current illness did not decrease the recovery of pathogens (9% with antibiotic use versus 6% without, p = 0.25). *Streptococcus pneumoniae *was the most common bacterial isolate, recovered in 52 blood cultures among 51 patients, accounting for 58% of all pathogens and 4% of all blood cultures. (One patient had bacteremia with serotype 6B pneumococcus in February and August 2007.) Nontyphi *Salmonella *group B accounted for 15 (17%) isolates, *Salmonella *Typhi for 9 (10%) and *Staphylococcus aureus *for 8 (9%). The remaining isolates included two *Escherichia coli*, and one each of nontypable *Haemophilus influenzae*, Group B streptococcus, and *Klebsiella pneumoniae*.

The median age of the 51 patients with pneumococcal bacteremia was 30 years (IQR 23-47); 11 (22%) were 5-17 years (Table 1). Children, 5-17 years were less likely than persons ≥18 years to have pneumococcal bacteremia (1.6% versus 6.9% of cultures, p < 0.001), and less likely to be infected with HIV (11% versus 82%, p < 0.001). Overall there was no difference by sex (52% female). Two patients (4%) had concomitant malaria parasitemia.

**Table 1:** Bacteremia by age group among persons ≥5 years of age presenting to Lwak Hospital, rural western Kenya, October 2006 - September 2008

Age in years	5-17	18-34	35-49	≥50	Total
Number patients	7683	3678	2375	2586	16322

Number blood cultures (% patients)	703 (9.2 )	275 (7.5 )	186 (7.8)	137( 5.3)	1301 (8.0 )

Positive blood cultures (% of cultures)^a^	21 (3.0)	33 (12.0)	22 (11.8)	13 (9.5)	89 (6.8)

Pneumococcus recovered (% of cultures)	11 (1.6)	22 (8.0)	10 (5.4)	9 (6.6)	52 (4.0)

Serotype 1 (% of pneumococci)	9 (81.8)	6 (27.3)	3 (30.0)	4 (44.4)	22 (42.3)

HIV positive/number tested (%)	1/9 (11.1)	12/13 (92.3)	4/5 (80.0)	2/4 (50.0)	19/31 (61.3)

^a^Excludes contaminants.

The majority (52%) of all pneumococcal bacteremias were among patients hospitalized with SARI. (Table 2). Likewise, the highest pneumococcal isolation rate from blood cultures was among patients admitted with SARI (10%), compared with 3% for SARI outpatients and for non-SARI admissions (Table 2). Of note, 6 of 9 admitted non-SARI inpatients had a cough or difficulty breathing, although not meeting the SARI definition due to lack of fever or low oxygen saturation. Using household surveillance data, four (7.7%) patients died within 30 days of their pneumococcal bacteremic episode, while another 3 (5.8%) died within the following 6 months (at days 30, 75 and 141).

**Table 2:** Bacteremia by clinical syndrome among persons ≥5 years of age presenting to Lwak Hospital, rural western Kenya , October 2006 - September 2008

	SARI^a ^- Not Admitted	SARI - Admitted	Admitted -- not SARI	Fever Only (temp >= 38.0)	Total
Number patients (N)	1030	418	822	481	2751

Number blood cultures (% clinic visits)	514 (49.9)	264 (63.2)	315 (38.3)	190 (39.5)	1283^b ^(46.6)

Positive blood cultures (% of cultures)^c^	25 (4.9)	41 (15.5)	18 (5.7)	4 (2.1)	88 (6.9)

Pneumococcus recovered (% of cultures)	14 (2.7)	27 (10.2)	9 (2.9)	2 (1.1)	52 (4.1)

Serotype 1 (% pneumococci)	6 (42.9)	11 (40.7)	3 (33.3)	2 (100)	22 (42.3)

HIV positive/number tested (%)	5/9 (55.6)	9/13 (69.2)	5/7 (71.4)	0/2 (0)	19/31(61.3)

^a^SARI is severe acute respiratory illness (see methods).

^b^18 patients were cultured for other reasons besides the 4 criteria listed.

^c^Excludes contaminants.

Among the 17 serotypes identified, serotype 1 accounted for 42%, followed by serotypes 6B and 15A (8% each, Table 3). Thirteen (25%) isolates were serotypes in the 7-valent pneumococcal conjugate vaccine (PCV7) and 37 (71%) were in the 10-valent conjugate vaccine (PCV10). Serotype 1 was more common among younger patients (82% among 5-17 year olds versus 32% among persons ≥18 years, p = 0.005), but did not vary by sex (Table 1). Among patients ≥18 years old with HIV status available, serotype 1 tended to be less common among HIV-positive patients (2/18, 11%) compared with HIV-negative patients (2/4, 50%, p = 0.13). The percentage of serotypes in PCV10 was lower among HIV-positive persons (12/19, 63%) than among HIV-negative persons (12/12,100%), p = 0.026).

**Table 3:** Serotypes of pneumococcal isolates from blood culture among persons ≥5 years of age presenting to Lwak Hospital, rural western Kenya, October 2006 - September 2008

Serotype	**# (%) of total (n = 52**^**a**^**)**
1	22 (42)
6B	4 (8)
15A	4 (8)
4	3 (6)
9V	3 (6)
3	2 (4)
5	2 (4)
13	2 (4)
19F	2 (4)
7A	1 (2)
7C	1 (2)
14	1 (2)
20	1 (2)
22F	1 (2)
23A	1 (2)
35B	1 (2)
43	1 (2)

PCV7 serotypes^b^	13 (25%)

PCV10 serotypes^b^	37 (71%)

PCV13 serotypes^b^	39 (75%)

^a^52 blood cultures yielded 52 serotypes among 51 patients. One patient had 6B bacteremia twice in February and August 2007.

^b^PCV7 includes serotypes 4, 6B, 9V,14, 18C, 19F, 23F. PCV10 adds serotypes 1, 5 and 7F. PCV13 adds serotypes 3, 6A, and 19A.

**Figure 1:**
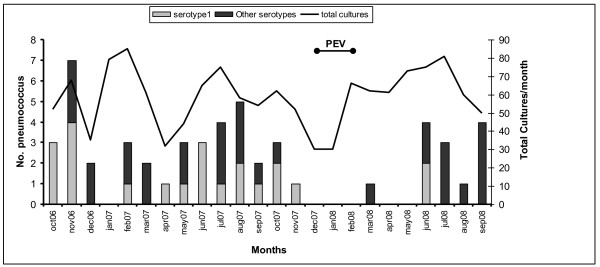
**Seasonality of pneumococcal bacteremia in rural western Kenya, October 2006 - September 2008**. PEV is post-election violence period when clinic utilization decreased.

There was no clear seasonal pattern to pneumococcal disease, nor did there appear to be temporal or spatial clustering of serotype 1 cases (Figure 1). There were no differences in syndrome when comparing serotype 1 to other serotypes (76% versus 81% were from SARI patients, respectively, p = 0.70). Among patients infected with serotype 1 pneumococci, 62% were hospitalized and 9.5% died, which was similar to those infected with other serotypes, where 74% were hospitalized and 6.5% died (p = NS).

Among 51 isolates with MIC testing, intermediate resistance was found among 1 (2%) isolate for parenteral penicillin, 2 (4%) isolates for amoxicillin and 1 (2%) isolate for cefotaxime; no full resistance to beta-lactams was found. Trimethoprim-sulfamethoxazole nonsusceptibility was high - 45 (88%) isolates were resistant and 4 (8%) were intermediate. Only 1 (2%) isolate was resistant to chloramphenicol and 1 (2%) other isolate was resistant to clindamycin and erythromycin. No isolates were nonsusceptible to levofloxacin.

The overall crude rate of pneumococcal bacteremia in persons ≥5 years was 129 cases per 100,000 person-years; the overall adjusted rate was 419 per 100,000 person-years (Table 4). Rates were highest among 18-49 year olds. As a comparison with adult bacteremia rates, during the same time period five pneumococcal isolates were recovered in children aged < 5 years in 6,932 person-years, yielding a crude incidence rate of 72 per 100,000 person-years with an adjusted rate of 203 per 100,000 person-years. The extrapolated rate of pneumococcal bacteremia among HIV-infected persons ≥18 years in the surveillance population was 2,399 per 100,000 person-years, compared with 122 per 100,000 person-years in HIV-negative persons ≥18 years old (Rate ratio 19.7, 95% CI 12.4-31.1). Extrapolating these rates to the Kenya population, we project that in 2010 there will be 58,483 cases of pneumococcal bacteremia among persons ≥18 years old in Kenya, 34,872 among HIV-positive and 23,611 among HIV-negative persons.

**Table 4:** Rates of pneumococcal bacteremia in rural western Kenya, October 2006 - September 2008

Age in years	*Syndrome*	*S. pneumo *(n)	**Pyo**^**a**^	Rate per 100,000 Pyo (95% CI)	% cultured	**Rate, Extrapolation 1**^**b **^**Pyo (95% CI)**	% clinic visits to Lwak	**Rate Extrapolation 2**^**c **^**Pyo (95% CI)**
5.17	Overall	11	17917	61 (31-110)	--	160 (65-255)		301 (123-479)
	*SARI Inpatient*	5			64		95	
	*SARI Outpatient*	4			47		54	
	*Admit, not SARI*	0			45		95	
	*Fever, not SARI*	2			38		41	

18-34	Overall	22	10869	202 (127-306)	--	391 (227-554)		578 (336-820)
	*SARI Inpatient*	10			56		80	
	*SARI Outpatient*	8			60		51	
	*Admit, not SARI*	4			36		79	
	*Fever, not SARI*	0			40		43	

35-49	Overall	10	4939	202 (97-372)	--	407 (155-659)		591 (225-957)
	*SARI Inpatient*	5			66		77	
	*SARI Outpatient*	2			55		52	
	*Admit, not SARI*	3			34		72	
	*Fever, not SARI*	0			62		42	

≥50	Overall	9	6738	134 (61-253)		232 (80-384)		354 (123-585)
	*SARI Inpatient*	7			71		67	
	*SARI Outpatient*	0			46		45	
	*Admit, not SARI*	2			35		63	
	*Fever, not SARI*	0			44		38	

Overall		52	40463	129 (96-169)		245 (178-312)		419 (305-533)

^a^pyo - person years of observation

^b^Extrapolation 1 accounts for patients meeting case definitions for blood culture in Lwak clinic who did not have blood cultures done (see methods).

^c^Extrapolation 2 uses the rates for extrapolation 1 and extrapolates further by accounting for the % of persons at the biweekly home visit with SARI or fever who went to a clinic other than Lwak clinic (see methods).

## Discussion

Like most studies of bacteremia in older children and adults in Africa, pneumococcus was the most common pathogen identified, accounting for 58% of isolates [1,4-9]. Because our study was population-based with active surveillance in the community, we could adjust crude rates to better assess actual rates of pneumococcal bacteremia, which was almost double that measured in the clinic. Moreover, home-based HIV testing in the community allowed calculation of rates of pneumococcal bacteremia in HIV-infected persons, which showed that almost one in four HIV-infected adults likely suffer pneumococcal bacteremia each year. The extrapolated rate of pneumococcal bacteremia among HIV-positive persons in our population (2,399 per 100,000 person-years) rivaled the crude rates among prospective cohorts of South African miners (2,136 per 100,000 person-years) and HIV-infected commercial sex workers in Nairobi (2,380 per 100,000 person-years) and HIV-infected adults in a trial of pneumococcal polysaccharide vaccine in Uganda (1,200 per 100,000 person-years), and was higher than the crude rate observed in urban South Africa (197 per 100,000 persons) [6,12-14]. In rural western Kenya, HIV infection seems to drive the high overall rate of pneumococcal bacteremia among adults, being 19.7 times more common among HIV-positive than HIV-negative adults; this elevated rate ratio is similar to South Africa (rate ratios of 6-55) and urban Kenya (rate ratio 17.8) [6,7,13-15].

The majority (79%) of pneumococcal bacteremic episodes occurred in patients with SARI with a predominance among those hospitalized. Only 5 (10%) of pneumococcal bacteremia patients did not report any respiratory symptoms. However, culturing only hospitalized patients would have missed 31% of pneumococcal bacteremia. Studies of invasive pneumococcal disease in Africa have yielded case-fatality proportions averaging 14-16%; the lower case-fatality proportion we found (7.7%) likely results from inclusion of outpatients, as well as not collecting cerebrospinal fluid on higher mortality meningitis cases [6,7,9,12,14,24]. The serotype distribution in western Kenyan is similar to that found in other adult populations in Africa [14,24-31]. Serotype 1 is uniformly the most common serotype found among African adults, ranging from 18-36% [28]. Serotype 1 predominated in large outbreaks of pneumococcal disease in the pre-antibiotic era and has more recently caused outbreaks of meningitis in West Africa [32,33]. We did not find evidence for serotype 1 outbreaks in our population as no temporal or spatial clustering of cases occurred. The increased prevalence of serotype 1 in HIV-negative compared with HIV-positive individuals, and the converse for the so-called pediatric serotypes in PCV7, has been shown before [14,15,26,31]. It has been postulated that HIV-infected adults, like young children, have reduced immunity to pneumococcal colonization, leading to increases in disease due to pediatric serotypes that commonly colonize the nasopharynx [26]. Indeed, nasopharyngeal colonization among HIV-infected adults has been shown to be elevated, with rates above 30% in Kenya and Zambia [34,35]. The high proportion of pneumococci resistant to cotrimoxazole has been shown before, and likely reflects widespread use of cotrimoxazole in the community and use of sulfadoxine-pyrimethamine for malaria treatment in Kenya [35]. Beta-lactam resistance remains low in Kenya and likely reflects the fact that apart from amoxicillin, few beta-lactam antibiotics are used outside of the inpatient setting.

In Kenya, several large-scale interventions that are being implemented could lead to a decrease in adult pneumococcal disease. First, until recently most Kenyans did not know their HIV status. The 2007 Kenya AIDS Indicator Survey showed two-thirds of Kenyans did not know their HIV status and 65% of HIV-infected persons who needed HAART were not receiving it [22]. However, HIV testing is now becoming more widespread in Kenya and HAART is increasingly available for those who need it due to large multinational funding initiatives. HAART has been shown to decrease invasive pneumococcal disease, as well as bacterial pneumonia, in HIV-infected persons [7,36]. Second, Kenya plans to introduce PCV10 (Synflorix™, GlaxoSmithKline, Rixensart, Belgium) for children in 2010 with support from the Global Alliance for Vaccines and Immunizations. In the U.S., a large indirect effect of childhood PCV7 (Prevnar™, Wyeth Pharmaceuticals, New Jersey, U.S.) vaccination was realized among adults, with a significant decrease in invasive pneumococcal disease of 32% among persons 20-39 years, the age group likely to be parents of small children and also that had the highest rate of pneumococcal bacteremia in our study [37]. After PCV7 was introduced in the U.S., HIV-infected adults showed a 62% decrease in pneumococcal disease due to vaccine serotypes [38]. PCV10 will include serotype 1, the most common serotype among Kenyan adults. Unlike most serotypes included in PCV7, serotype 1 is not often found in the nasopharynges of children [32]. Because the indirect impact of conjugate vaccines work through reduction of carriage, and thus transmission to other persons, whether the same degree of herd immunity in Kenyan adults will be realized for serotype 1 is unclear.

Our study had several limitations. First, we did not have HIV status on all patients and assuming the same HIV prevalence among those not tested might have been incorrect. In particular, patients who died before the home-based HIV testing initiative began might have been more likely to be HIV-positive, which would have resulted in even higher extrapolated rates of pneumococcal bacteremia in HIV-infected persons than we calculated. Due to the small number of patients with pneumococcal bacteremia who had HIV testing in our study, the HIV-specific results should be interpreted with some caution. Moreover, our projection of the national number of pneumococcal bacteremia cases among adults assumed that no persons ≥65 years old were HIV-positive, so likely underestimated the true number of cases. Second, in making extrapolations of bacteremia rates, we made some assumptions that could have biased our adjusted rates. We assumed isolation rates would be the same among those with the same syndromes who did not get blood cultures. At Lwak, cultures might have been performed more frequently among patients more likely to have pneumococcal bacteremia (e.g. sicker patients). Also, persons in the community might have been more likely to go to Lwak than other clinics if they were sicker, knowing that admission was free at Lwak. Third, we only cultured the blood of the first two febrile adults, which possibly could have led to a systematic bias if the first two patients were more or less likely to have bacteremia than other febrile patients. Moreover, by culturing the majority of SARI patients, while only a sample of febrile patients, we likely biased our results towards finding more pneumococcus and less mycobacteria and non-typhi salmonellae, which have also been shown to be prevalent, sometimes more so than pneumococcus, among febrile, HIV-infected persons admitted to hospital [4,9-11]. Lastly, because we did not do lumbar punctures, our analysis did not include pneumococcal meningitis, the other major manifestation of invasive pneumococcal disease.

## Conclusions

We showed a high burden of pneumococcal bacteremia in older children and adults in western Kenya. As shown before, that burden is exacerbated by the HIV epidemic in Africa. Further surveillance is needed to assess the impact of potential preventive strategies, such as expanding HAART among HIV-infected persons and the indirect effect of the introduction of pneumococcal conjugate vaccines in children, and to decide whether additional targeted interventions, such as adult vaccination with pneumococcal conjugate vaccine, might be needed to alleviate the great burden of pneumococcal disease among older children and adults in Africa [39]. Lastly, in light of the high rate of bacteremia that we and others found in adults in parts of Africa with high HIV prevalence, more widespread provision of microbiology services in clinical settings could result in improved patient care.

## Competing interests

The authors declare that they have no competing interests.

## Authors' contributions

DF conceived of study and oversaw data analysis and drafting of manuscript. GJ took the lead on microbiologic work at KEMRI/CDC and assisted in data analysis. BA was the lead data analyst. GB coordinated the clinic and field aspects of data collection. JO set up blood culture capacity and oversaw the microbiology lab at KEMRI/CDC in Kisumu. BB oversaw serotyping and antibiotic susceptibility testing at CDC in Atlanta. AK and SM performed serotyping at KEMRI/Wellcome-Trust labs in Kilifi. MKN is the director of the International Emerging Infections Program at KEMRI/CDC and oversaw all lab work for this project. RB conceived of the surveillance and oversaw the project. All authors read and approved of the final manuscript.

## Pre-publication history

The pre-publication history for this paper can be accessed here:

http://www.biomedcentral.com/1471-2334/10/186/prepub
